# Stakeholder views of managed entry agreements: A literature review of national studies

**DOI:** 10.1016/j.hpopen.2021.100032

**Published:** 2021-01-21

**Authors:** Subramaniam Thanimalai, Wai Yee Choon, Kenneth Kwing-Chin Lee

**Affiliations:** a School of Pharmacy, Monash University Malaysia, Bandar Sunway, Malaysia; b School of Pharmacy, Monash University, Malaysia

**Keywords:** Managed entry agreement/ arrangements, Patient access scheme, Reimbursement, Stakeholder, Review

## Abstract

Access to highly expensive and innovative medicine is a challenge. Managed entry agreements/ arrangements (MEAs) consist of various forms of confidential agreements between pharmaceutical manufacturers and payers (hospitals, social insurance), which are mainly negotiated when there is uncertainty of the actual clinical benefit of the medicines, but high public expenditures are required. Continued and expanding use of these schemes requires a greater understanding of payer perceptions, experience, and orientation towards their current and future use. This literature review aimed to identify the stakeholders’ concerns with regards to MEAs. A total of 20 articles were identified between 2007 and 2018. MEAs are mostly studied in the first world countries, whereas limited documentation is available for other nations. The reasons for rejection of proposed MEAs could possibly be related to existing low-cost effective treatments, lack of trust between stakeholders, high administrative burden and if the authorities believe they are funding the substantial proportion of the drug’s development cost. Lack of well-designed and easy-to-use data collection methods for MEAs has an impact on the evidence generation. Most articles highlighted the lack of transparency of the agreements which may not benefit other health systems, especially for systems that are dependent on international referencing price. Stakeholders also identified the lack of expertise in assessing the pharmacoeconomic and health outcomes data and challenges in assessing risks up-front due to the complexity of the real-world data. Lack of transparency in most health systems are also a hindrance to further understanding and confidence in the MEAs. However, the views of the stakeholders may also evolve with experience. Though there are challenges in the designing and implementation of MEAs, close networking, improved human capital development especially in the area of health technology assessment and health economics would be able to close the gap in the implementation.

## Highlights

• Rising cost of innovative pharmaceuticals may affect access.
• Uptake of managed entry agreements/ arrangements are slow.
• Challenges in implementing the schemes needs to be addressed to improve access.
• Engagement of stakeholders will bring more light to the challenges faced.

## 1 Introduction

Well-functioning health systems are of critical importance to the achievement of both national policy objectives and international policy commitments, such as the Millennium Development Goals [MDGs] of the United Nations [1]. Mixed public-private finance is widespread in health systems internationally. Public finance predominates in most countries, while private finance plays a variety of roles that depend on the design of the financing system [2]. Health financing involves not only methods of raising money for health care, but also efficient allocation of those funds. There is competition for funds in any system, and how money is allocated affects not only the way the services are provided but also setting of priorities. The allocation of resources requires a skilful planning process to balance spending on different subsectors of the system and to ensure equity between regions and various socioeconomic groups in society [3].

Policymakers face triad of challenges when implementing pharmaceutical policies that aim to achieve affordable, equitable and at the same time, sustainable access to medicines. Reimbursement policies for medicines should not be considered in isolation as there is a strong link between pharmaceutical pricing and reimbursement. A key reimbursement instrument to ensure affordable patient access to medicines is a reimbursement list that specifies medicines eligible and selected for coverage (positive list) or explicitly excluded from reimbursement (negative list). Eligibility for reimbursement coverage may depend on the medicine (product-specific) or the disease the medicine aims to treat (disease-specific). It may also be linked to a specific population group in need of medicines (population groups-specific) or the total medicine expenditure of a patient within a certain time-period (consumption-based). Uncertainties regarding the clinical evidence, cost-effectiveness or budget impact of medicine may prevent health care payers from reaching conclusions on coverage decisions, thus affecting patient access [4]. Under the current institutional framework, the growth of pharmaceutical expenditures due to new high-cost innovative medicines, creates financial challenges to health systems. The recognition that the current path of growth cannot be continued indefinitely leads to the search for new ways to ensure that innovation “that matters” is produced, where patients have access to innovation and that health systems are financially sustainable [4].

National healthcare payers have been increasingly looking into innovative reimbursement approaches to balance the need to provide rapid access to potentially beneficial health technologies to patients with the requirements to obtain the best value for money and to ensure affordability [5]. Several arrangements to set prices and access conditions for new medicines have been experimented by some national authorities in charge of pricing and reimbursement decisions. These include value-based pricing and managed entry agreements. “Value-based pricing” stands for the assessment of the therapeutic value of medicines and the according pricing deduced from the clinical value. Managed entry agreements or arrangements (MEAs) consist of various forms of confidential arrangements between pharmaceutical manufacturers and payers (hospitals, social insurance) that are subsumed, which are mainly negotiated when there is uncertainty of the medicines actual clinical benefit but high public expenditures are required [6].

MEAs are typically divided into finance- or outcomes/ performance-based arrangements. MEAs may reduce uncertainty about the real value of medicines, though meaningful additional data (real-life data) are collected under those arrangements but they are not necessarily published. They are also expected to prevent complete exclusion from reimbursement due to the uncertain clinical benefit and to keep the budget under control. They The MEAs anticipate access to the new product at the cost of delaying some steps of the standard process for taking the new products to the patients. The strong points of MEAs are different for distinct stakeholders (health care payer, patients, companies), as each focus on a different main objective (e.g, budget control, access, obtaining reimbursement with a non-disclosed price) [6]. Continued and expanding use of these schemes requires a greater understanding of payer perceptions, experience and orientation towards their current and future use [7]. This review aimed to identify the stakeholders’ concerns with regards to MEAs. A literature review was performed to identify the concerns of various country stakeholders, as most studies had varied methodology such as semi-structured interview, online survey or in-depth interview and analysis methods.

For this literature review, the phenomenon of interest is MEAs or patients access schemes used to improve access to high-cost medicines. MEAs covers various reimbursement methods used to improve access to high-cost medicines. These reimbursement methods include both finance- and performance/ outcomes-based arrangements between the healthcare service provider/ payer and pharmaceutical company. Views of healthcare personnel, pharmaceutical company personnel, and others (e.g. patient advocacy group, health service and insurance providers) are also included in this review.

From the literature, it was observed that the policymakers are involved from wide stakeholder groupings. This included both the macro and micro levels. Macro level includes international/ national leaders, while micro level includes physicians, formulary pharmacists, and representatives of government, hospital executives, ethicists, administrators and pharmacy managers [8], [9]. Internal frameworks and personal values of decision-makers have been reported to be in contrast with decision criteria and may influence the approach by decision-makers. Evidence also suggests that equity of access to high-cost medicines amongst different patient groups, particularly of biologicals is difficult. Viewpoints of stakeholders and barriers to access could be explored at a different levels [10].

This literature review aims to identify, appraise and synthesise qualitative research evidence on the stakeholders’ perceptions and experiences on MEAs, to access high-cost medicines. In this review, we have also identified hypotheses for subsequent consideration and assessment of MEAs.

## 2 Method

### 2.1 Criteria for considering studies for this review

#### 2.1.1 Types of studies

We included primary studies that used qualitative methods for data collection (e.g. interviews, focus group discussions, document analysis and observations) related to managed entry arrangements/ patient access schemes, and/or qualitative methods for data analysis (e.g. thematic analysis and grounded theory). We also included primary studies that collected data using qualitative methods but did not perform a qualitative analysis (e.g. online survey, open-ended survey, questionnaire where the responses are analysed using descriptive statistics). Mixed methods studies were included when it was possible to extract data that resulted from the qualitative methods.

#### 2.1.2 Types of participants

##### 2.1.2.1 Participants

We included studies that focussed on the perceptions and experiences of personnel with roles in decision making and implementation of MEAs from the following categories:

• Public/ private healthcare professionals involved in the decision making or implementation of MEAs (clinicians (different clinical disciplines), pharmacists (different roles).
• Pharmaceutical company personnel
• Others; advisors, administrators, health service/ insurance managers, patients, patient advocacy group members, non-governmental organization members

#### 2.1.3 Settings

The MEAs could be implemented in public or private healthcare facilities, and/or in the community healthcare setup. We included studies conducted in any country.

#### 2.1.4 Phenomena of interest

The phenomena of interest were managed entry arrangements/ patient access schemes related decision making and its’ implementation, stakeholders’ view of implementation, challenges and general views of the MEAs.

### 2.2 Search methods for the identification of studies

A structured search strategy, which combined potentially relevant keywords and controlled vocabulary (MeSH) terms, were first developed. The various reimbursement methods to improve patient access to medicines were grouped into Managed Entry Arrangements (MEAs). Thus, all the following terms were used to describe MEAs:

‘finance-based agreement’, price volume agreement’, ‘discounted treatment’, ‘performance-based agreement’, performance-linked coverage’, ‘outcome guarantee’, ‘money-back guarantee’, ‘conditional treatment continuation’, coverage with evidence development’, ‘managed entry agreement’, ‘risk-sharing agreement’, ‘risk-sharing scheme’, ‘patient access scheme’, ‘performance based risk sharing’, ‘health impact guarantee', 'controlled access’.

The keywords were combined and adapted to search databases. To ensure optimal coverage, additional articles were found within the reference section of retrieved articles and through citation snowballing, wider searches were undertaken.

**Fig. 1:**
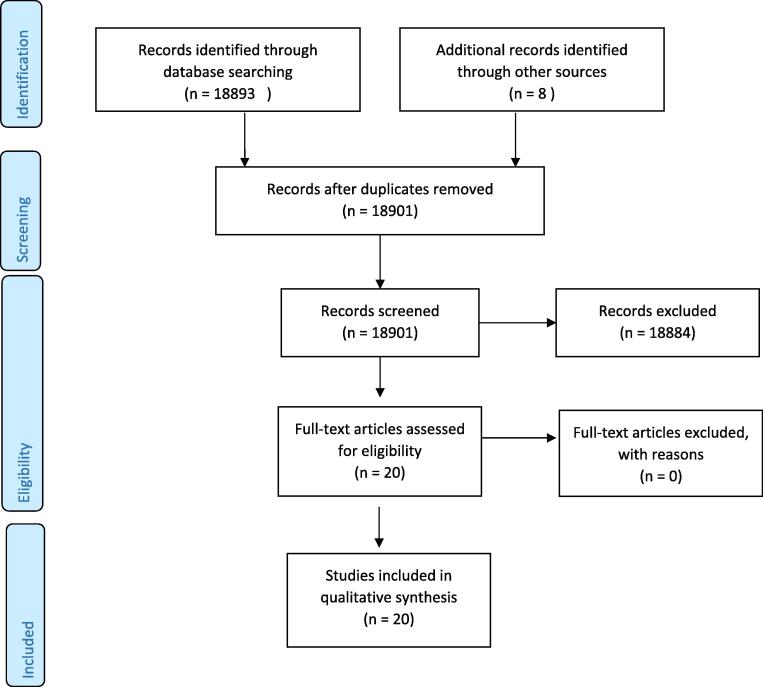
Abstract identification flow diagram.

Literature search and documentation of data was performed to collect information on MEAs. All the terms mentioned earlier were used for the search. 18,893 articles returned for the search term. Abstract review of the articles identified a total of 21 articles with stakeholders’ views of the MEAs. 1 was a Spanish article with only an English abstract. Eventually, 20 full English articles were identified for the literature review (See Fig. 1).

The title and abstract of all retrieved articles were reviewed by the lead author (ST). Articles that met the inclusion criteria (Table 1) were retrieved and examined more closely by the second author (JC) in conjunction with the review. The quality of research papers was evaluated according to an adequate description of the theoretical framework, background and methodology.

**Table 1:** Characteristics of included studies and quality assessment.

Author/ Year	Country	Treatment focus	Number of participants	Type of participants	Methods
Lu CY et al./ 2007	1 Country/(Australia)	Anti-rheumatology biologicals	36	Rheumatologist, patients with RA, government advisors, consumer advocates, public servant and pharmaceutical company spokesperson	Semi structured interview (in-depth interview), purposeful sampling
Jakub Adamski et al./ 2010	15 countries/(Sweden, Italy, UK, Germany, Estonia, USA, France, Spain, Lithuania, Australia, Belgium, Hungary, Portugal, Canada, Denmark)	Any	16	Health Authority Personnel from 14 different countries.	Literature review and combined knowledge of the personnel
Espin J. et al./ 2011	18 Countries (Austria, Denmark, Finland, Iceland, Ireland, Italy, Lithuania, Malta, Norway, Poland, Portugal, Romania, Slovenia, Spain, Sweden, UK)	Oncology	19	Members of the Network Meetings of the Competent Authorities on Pricing and Reimbursement EU and EFTA countries	Online Survey(First – on Oncology products and Second on one Risk Sharing Scheme in each country)
Sabine Vogler et al./ 2012	20 EU Member states and 6 non-EU	Any	26	Members of PPRI member network (Medicines Agency and Ministry of Health)	Open ended questionnaire sent via e-mail and review of information
Lisbet Coulton et al./ 2012	Australia, Taiwan, Indonesia, Singapore, Mainland China, Philippines, Malaysia, South Korea, Thailand	Any	14	Expert panel; Consultants, Academics, Researcher, NGOs, Pharma personnel	Review of information, Expert panel discussion and detailed questionnaire
Ariel Hammerman et al./ 2012	Israel	Any	31	Government Official, Senior Managers of Health Plan, pharmaceutical industry executive, academic researchers, Non-Governmental organization	Semi-structured questionnaire, face to face interview, audio record and transcribed verbatim, Purposive sampling
Paige A. Thompson/ 2012	Canada	Any	9	Drug Plan Managers – Canadian Provincial Government representatives	Literature review and key informant interview using semi-structured questionnaire via telephone, Purposive sampling
Steve G. Morgan et al./ 2013	Canada	Any	9	Senior Executive of Health plan	Semi-structured questionnaire, Telephone interviews with purposefully selected individuals
Louis. P Garrison et al./ 2015	U.S	Any- Private sector	14 (interview), 15 (online survey)	Pharmaceutical company, Payer organization and industry experts	Review of database, Key informant interview – in-depth 1 h telephone interview and online survey. Purposive sampling
Melissa Thompson et al./ 2016	Canada	Any	21	HTA key opinion leaders, pharmaceutical industry chief executive officer/vice presidents, ex-payers, current payer/drug plan managers	Telephone or in-person interviews (5-questions version or a 16-questions version)
Arvind Mani et al./ 2016	Canada	Any – Private Sector	30	Pharmaceutical companies and payer representatives	Online survey
Taza Nazareth et al./ 2017	US, France, Germany, Italy, Spain, UK	Any	27	Senior experts from US and EU-5 national payer, pharmaceutical manufacturer, pricing and market access executive	Structured interview with targeted literature review. Telephone interview with senior experts (1hr)
Kim Pauwels et al. / 2017	Belgium, the Netherlands, Scotland, England & Wales, Sweden, Italy, Czech Republic, France	Oncology	Not stated	Authorities involved in set-up and negotiation of Managed Entry Agreements and PPRI network members	Literature search & document analysis, Semi-structured interview via telephone and audio recorded and verbatim
Steve. G Morgan et al./ 2017	11 developed countries (Australia, Austria, Canada, England, Germany, New Zealand, Norway, Scotland, Sweden, the Netherlands, USA)	Any	10	Public or social health systems – Manager & technical experts	Anonymous online survey
Joseph A. Goble et al./ 2017	US	Any	32 responses (web-survey)	Payer Medical and Pharmacy Directors	37 item questionnaire, web-survey
Alessandra Ferrario et al./ 2017	16 (Latvia, Poland, Bosnia & Herzegovina, Hungary, Slovenia, Lithuania, Albania, Kosovo, Estonia, Romania, Russia, Serbia, Bulgaria, Poland, Slovakia, Croatia, Czech)	Any	16	Senior staff in competent authorities for pricing and reimbursement, academics with expertise on National pharmaceutical issues, national experts in pharmaceutical matters, Members of Piperska group	Questionnaire Survey send to informants
William C.N. Dunlop et al./ 2018	France, Germany, Italy, Spain, the UK	Any	66	Hospital pharmacist with procurement responsibility, current or former members of regional or national healthcare payer or budget-setting organisations	Online survey (web based with closed, multiple choice questions) for stakeholder
Christiane Maskineh et al./ 2018	Middle eastern and northern African region (Algeria, Egypt, Lebanon, Jordan, United Arab Emirates (UAE), Kingdom of Saudi Arabia (KSA))	Any	44	Public sector, pharmaceutical industry	Prospective, cross-sectional, mailed online survey
Jacoline C. Bouvy et al./ 2018	Europe (Spain, Italy, Germany, France, Austria, UK, Norway, Sweden, the Netherlands)	Any	Semi-structured interview – 17 Questionnaire – 13 Workshop − 30	Manufacturer, payer, HTA agency personnel	Semi-structured questionnaire (face to face or telephone) and questionnaire to identify status of products. Results discussion during workshop
Alexandru M. Rotar et al./ 2018	6 (Bulgaria, Croatia, the Czech Republic, Hungary, Poland, Romania)	Any	10	Country Experts	Questionnaire and extended phone calls

Stakeholders were defined in this review through the application of the definition of Lu CY et al. [11] as “individuals or groups of people that have the potential to influence the decisions on the access”. Decision-makers are a heterogeneous group consisting of health policymakers, insurance managers, committee members, hospital managers and others.

## 3 Findings and Discussion

### 3.1 General findings

The number of publications on the stakeholder’s views of MEAs has constantly increased over the years, with most papers between 2017 and 2018. This shows a growing interest in the area of improving patient access to innovative medicines and the role and impact of stakeholder views on MEAs implementation. The stakeholder views are closely related to the implementation of the MEAs in their countries.

### 3.2 Geographic distribution and diversity

Most studies were based in Australia, Europe and Northern America. These studies covered various MEAs, different stakeholders and different disease areas. As for other countries, one study assessed the views of the Israeli [12] and another the Middle Eastern and North African [13] region stakeholders. Only one study covered the Asia Pacific region, covering mostly Australia, Taiwan, Indonesia, Singapore, Mainland China, Taiwan and Philippines. This article also had a brief mention of Thailand and Malaysia [14]. MEAs were commonly implemented for antineoplastic and immunomodulating agents, haematological agents, alimentary tract and metabolism, nervous system, blood and blood-forming agents, endocrine agents, anti-infectives for systemic use and other therapeutic agents. Limited evidence of MEAs were also available for cardiovascular, respiratory, sensory organs, genitourinary and sex hormones, musculoskeletal, ophthalmological and anti-parasitic products [11], [15].

The study characteristics are presented in Table 1. The MEAs stakeholder related studies were identified between the years 2007 and 2018. The studies mostly used qualitative or mixed methodology to elicit information on stakeholders’ opinions. Most of the studies sought views for all types of MEAs, except for three articles, where two focussed on oncology products [15], [16] and another on anti-rheumatic biological products [11]. Multiple methods were used to assess stakeholders’ views and information on MEAs. This review is based on 496 respondents from the selected articles, where 210 were online survey respondents, 81 were expert panel interviews, 71 responded via face-to- face interviews, 62 responded via mail/e-mail questionnaires, 42 provided an in-depth interview and another 30 responded via a workshop. Thirteen studies used a single method to assess stakeholders’ views, where five studies used an emailed questionnaire [7], [17], [18], [19], [20], three used an online survey [13], [21], [22], while the other five used structured/semi-structured interviews [11], [12], [19], [23], [24]. The rest of the studies used a combination of two or more methods such as literature review, expert panel discussion, expert opinion/ interview and expert workshop, online survey, questionnaire or structured or semi-structured questionnaire [14], [15], [16], [25], [26], [27], [28], [29]. The number of respondents for each study ranged between 7 and 66. The most used method to obtain stakeholders’ views were semi-structured questionnaire, questionnaire or an online survey.

The types of stakeholders identified in the studies are presented in Table 2. The most included stakeholders were public servants, pharmaceutical organization personnel and health plan managers. Limited studies used government advisors and patientsadvocacy groups, while patients’ views were only sought in an Australian study.

**Table 2:** Types of stakeholders whose views were sought for the implementation of MEAs.

Author/ Year	Government/ Public service personnel	Manufacturer/ Pharmaceutical organization	Health plan manager/ Health insurers	Clinicians	Pharmacist (Various role)	Academics	Consumer advocates/ non-governmental organization members	Government Advisors/ Experts/ Health technology assessment agency	Patients
. Lu CY et al./2007	✓	✓		✓			✓	✓	✓
Jakub Adamski et al./2010	✓		✓	✓	✓	✓		✓	
Espin J. etal./2011	✓								
Sabine Vogler et al./2012	✓								
Lisbet Coulton et al./2012	✓	✓			✓	✓	✓	✓	
Ariel Hammerman et al./2012	✓	✓	✓	✓		✓			
Paige A. Thomson et al./2012			✓						
Steve G. Morgan et al./2013			✓						
Louis. P Garrison et al./2015		✓	✓					✓	
Melissa Thompson et al./2016		✓	✓					✓	
Arvind Mani et al./2016		✓	✓						
Taza Nazareth et al./2017		✓	✓						
Kim Pauwels et al./2017	✓		✓						
Steve G. Morgan et al./2017	✓		✓						
Joseph A. Goble et al./2017			✓	✓	✓				
Alessandra Ferrario et al./2017	✓		✓			✓	✓	✓	
William C.N. Dunlop et al./2018			✓		✓			✓	
Christiane Maskineh et al./2018	✓	✓							
Jacoline C. Bouvy et al./2018		✓	✓					✓	
Alexandru M. Rotar et al./2018								✓	

Most of the studies described the types of questions and type of information which were sought in the study. The question domains and type of questions were only described in one study [21]. The domains defined in the study were respondent characteristics, disease areas, drug and disease attributes, accessibility of data, mechanisms for payment and reimbursement, critical components, payer satisfaction/ dissatisfaction, manufacturer and third-party facilitation and contract templates. The questions used in most of the studies assessed the objectives [12], [14], [15], [20], [23], [26], [28], strengths/ weaknesses [7], [11], [12], [13], [14], [16], [19], [21], [22], [23], [25], [26], [27], [28], types of schemes [4], [13], [14], [15], [16], [17], [18], [19], [20], [21], [25], [26], [27], contract framework [11], [12], [14], [15], [17], [18], [20], [21], [22], [24], [25], administrative burden [12], [14], [19], [20], [21], [25], [27] and its' future implementation [7], [12], [13], [14], [22], [26], [27], [28]. Some of the less common questions addressed issues related to the role of respondents in the design of MEAs, choice of decision-makers who should be involved in the MEAs related decision making, individual roles and responsibility of health and pharmaceutical organization personnel in implementing MEAs, type of changes or improvement noted, monitoring of MEAs and its’ impact.

The stakeholder views were sought for all types of MEAs, except for 7 articles that focussed on outcomes/performance- [14], [21], [27] or finance- [12], [17], [19], [23]based MEAs specifically. The stakeholder studies addressed the area of concerns with regards to the planning and implementation of MEAs.

### 3.3 MEAs objectives

Stakeholders in finance-based MEAs article stated that the strategy was executed to create expenditure and drug budget certainty. Finance-based MEAs for specialty pharmaceuticals were meant to lower costs. The mechanism was seen as a way to improve budgetary balance of the health plans [12], [19], [24]. Most stakeholders opined that the MEAs were meant as resource rationing, mainly to provide equitable or feasible care within a finite budget. Reducing the cost pressure in terms of price reduction allows coverage of maximum number of patients and certainty of drug budget.

As for outcomes-based MEAs article, the stakeholders felt patient gained early or improved access to innovative medicines. The schemes were also believed to help drive towards better disease management by improving the standards. The stakeholders felt it addresses the areas of high clinical unmet needs, where the disease affects a small patient population or where limited patient numbers have made trial data and data collection is generally more difficult; or where market access is restricted given the uncertainty at launch. These MEAs allowed the payers and patients to gain experience with the medication. Uncertainty of the clinical data can also be addressed during the data collection of the real-world scenarios, which is a common ground for pharmaceutical companies and payers. The manufacturers stated that they would use the outcomes-based MEAs to differentiate and demonstrate the effectiveness of their product versus competitors [14], [26].

Most articles included both finance- and outcomes-based MEAs, where the objectives for implementation were similar. The payers reported that MEAs implementation were to limit total budget impact, to provide early access, to reduce uncertainty and to provide a technology that demonstrates value. Stakeholders felt the MEAs could provide access to expensive medicines through a publicly funded system. Targeting the access for patients with the most need for treatment was identified as spending “for value”. MEAs is also seen as a policy tool for the rationalization of introduction and use of pharmaceutical products. It is also believed to toaddresses the uncertainty on clinical effectiveness and/ or cost-effectiveness and manage utilization. When long-term outcomes have not been demonstrated by the pharmaceutical company, MEAs can reduce the risk to the payers while ensuring early access to innovative technologies. Changes in policy framework in some countries in Europe and Canada have also made the MEAs a regular part of coverage. Some jurisdictions used it to generate additional evidence on which price and/or reimbursement should be established. MEAs have also been a choice of reimbursement method if there is an unfavourable cost-benefit ratio, higher average price or above the willingness-to-pay. Certain jurisdictions claimed the political pressure as reasons for the implementation of MEAs. The pressure came from political spheres, as politicians receive pressure from interest groups. In addition to improving access and finance, MEAs addresses political pressures and enhances the public image of the stakeholders. Limited number of jurisdictions use it to either reimburse high-cost cost-effective medicine or for products which is unable to be made cost-effective due to the international reference pricing.

Working within the budget will ensure the sustainability of the health system. As for the pharmaceutical company, it can gain a competitive advantage, increase revenue and funds for further innovation and improve the image of the company.

### 3.4 Benefits

Despite the challenges in the implementation of the MEAs, many benefits of its' implementation were identified. The main benefit highlighted by the stakeholders were price reduction which provided budget predictability [15], [16], [19], [22]. In Europe, individual negotiations were identified to be able to bring a reduction between 0 and 50 % in the price, whereas price reduction laws/ regulations brought down prices by 3 to 32.5%, while refunds and payback mechanisms showed a 1 to 8 % reduction in sales value [17]. Limited evidence was available for discounts of more than 60% [19], [20]. The reduction in price lead to increased number of patients being covered [16]. The stakeholders also felt, use of MEAs as a tool for improving the accuracy of budget-impact estimations have not been examined. The MEAs implementation process led to improved clinical, financial and administrative documentation [11]. Some of the MEAs implementations have led to more up-to-date data documentation and collections methods which allows a more systematic treatment [11], [16], [25]. A more integrated evidence generation would lead to better decision making [28]. The increased communication between the stakeholders was also expected to improve collaborations. The collaborative work will also promote shared understanding for better management of resources and the healthcare budget. Resource constraints could be addressed with the introduction MEAs, as it provides different ways to manage the funding of new drugs, such as creation of Sickness Fund, the return of rebates and pay-for-performance. Parallel importation could be prevented as MEAs would offer a better price. It is also a positive sign for new products in terms of early access to the market. Minus the up-front investment for the negotiations and system development, MEAs are expected to provide cost and budget predictability and systematized the treatment. MEAs addressed the unmet needs and provides an expansive formulary. The stakeholders felt that the manufacturers were able to gain access, improve sales and value proposition. The MEAs has also met the political challenge with improved access to innovative treatment and ensuring that it is beneficial to patients [14].

### 3.5 Challenges

The question whether the objectives of the scheme is fully explicit and whether the level of evidence is enough to make robust decisions initially, hangs over the implementation of MEAs [25]. The stakeholders also highlighted the restrictive nature of the selection criteria in the implementation of the MEAs. The selection criteria had to be carefully selected to represent the right population and to ensure the financial implications are within financial means. The need for education on the application of the criteria appropriately and the risk of too restrictive criteria was also identified [11], [25]. Without the criteria or criteria unsupported by published literature, the payers run a risk of excessive uptake [11]. Patients were also apprehensive that an effective treatment would be withdrawn if one missed the threshold or response to qualify for the treatment [11]. The everchanging disease and pharmaceutical landscape requires the MEAs to be regularly reviewed and refined to ensure its success. The implementation of the MEAs required greater resources depending on the type of MEAs [11]. The reliance on the physician and the bureaucratic red tapes were sometimes considered a deterrent to the MEAs implantations [11]. A balance between practitioner/ personnel integrity and bureaucratic requirement is needed in the development and use of these criteria [11]. Limitations of use to sub-specialised clinicians and enrolment criteria needs be discussed with the relevant stakeholders for improved implementation. In addition to stakeholder engagement, education of physician and patients are also an integral part of the implementation. However adverse patient selection and uncontrolled use were not of a major concern among certain stakeholders. Manufacturer were also concerned about population based agreements as they are perceived as too risky because there are many unknowns around compliance, prescribing and so forth [26].

The other challenges highlighted were the high administrative burden, resource demands and costs to execute and manage the agreements. Siloed nature of the policies and capabilities, difficulty in identifying suitable clinical outcomes were also among the major concerns [24]. Though the use of finance-based schemes were simpler, it was viewed as a missed opportunity to leverage evidence generated post-approval [28].

Involvement of consumer organizations and patients were also a concern among stakeholders. Though the doctors were expected to be able represent the patients, a more direct role was preferred. Careful selection of patients was needed for process improvement, if not the initial stage, they could be involved at least at later stages of implementation. Consumer organizations were identified as having a role in disseminating information and providing consumer support. Both prescribers and nurses were concerned about the considerable workload imposed on them. In addition, the prescribers were uneasy there was no recompense for the substantial additional time to undertake the tasks [11]. Some policy makers felt they were powerless in the decision to use MEAs, as it has been thrust upon them by manufacturers, politicians and patient advocacy groups [23].

The stakeholders concern was that the payers considered the use of MEAs due to the refusal of the manufacturers to decrease the list price. This global phenomenon is due to the reference pricing [13], [17], [22], [23]. The lack of transparency in the drug price and the selection criteria, currently constrained by confidentiality requirements is considered essential to enhance the understanding of the system and enable payers to better defend the decisions Though it is leading to asymmetry of information, manufacturers have clearly identified MEAs as a desirable tool [11]. The concerns from the payer perspective were that the pharmaceutical organizations were benefiting from unproven drugs as the level of evidence was insufficient and the MEAs offers were aimed at gaining market shares. Some stakeholders highlighted the tendency of the pharmaceutical organization to submit prices that exceed the cost-effectiveness threshold. The opportunity cost of implementing the MEAs is also rarely explored [25]. Challenges were also noted at the initial stage of arriving at an agreement and at the renegotiation stage, which could delay listing of pharmaceuticals and their continued use [23].

Factors enabling or hindering the implementation

The reasons for rejection of proposed MEAs could possibly be related to existing low cost-effective treatments, high administrative burden and if the authorities believe they are funding the substantial proportion of the drug’s development cost [25]. Some health systems like Turkey charged manufacturers for various costs associated with the negotiation and implementation of MEAs. Many respondents also responded positively towards reimbursement of the cost of contracted drug failures, the adverse drug events and the alternative drugs.

Given the complexity of the MEAs, it must be also evaluated by the trained personnel [25]. The availability and reliability of the data were also a concern in most studies [25]. Many MEAs are designed based on the registry data, and there is a need to ensure the registries are reliable. The lack of data was mostly related to the increased workload among the existing workforce, the capacity of the existing medical records to produce the relevant information and on who should take up the responsibility of funding the evidence generation. Lack of well-designed and easy-to-use computer systems for MEAs has an impact on the evidence generation. Availability of good quality data is important to ensure there is consistency between clinical trials and the real-world population. Legal requirements surrounding the data protection were not widely available. Mixed health systems and structure, which is fragmented lead to difficult implementations of the MEAs. The need for coordination of the mixed providers exists. There was also little consistency in the role of health technology assessment bodies. Most articles highlighted the lack of transparency of the arrangements which may not benefit other health systems, especially for systems that are dependent on international referencing price. Larger health systems are also bound to receive larger discounts. The other important views were regarding the timely communication of the data or outcomes to the stakeholders. With largely varied MEAs schemes, the need for different payment mechanisms also exists. Standardised contract template to facilitate execution and transparent financial risk evaluation and modelling were highly ranked on how manufacturer can improve the implementation. Many agreed that bureaucracy could be administratively burdensome for all stakeholders.

Perceived value drivers and its' difference observed from both payers and manufacturer perspectives were an important determinant of its implementation. Though both payers and manufacturers agree that it reduces the cost for payers, there are differences in terms of improved patients’ outcomes. As for value for manufacturers, both parties agree on its improved level of access, however, payers were concerned it was increasing the reimbursement levels without much reduction in uncertainty [27]. Inter-jurisdictional MEAs are seen to improve bargaining power though it may bring lesser benefit to the partners who consume less. Inter-jurisdictional arrangements may also take longer period to be formalized and be more challenging in terms of monitoring the outcomes. Coordinating multi-country data collection efforts rather than seeking country-specific models could be initiated through early dialogue and might facilitate outcomes-based agreements. Simple models rather than complicated ones with simple data collection efforts involving the collection of few but essential parameters that are normally tracked by the health care system might be easier to implement, less costly and more feasible for coordination. The financial risk from the overuse or unexpected increase of the newly listed technology must be appraised up-front.

Stakeholders also identified the lack of expertise in assessing the pharmacoeconomic and health outcomes data and challenges in assessing risk up-front due to the complexity of the real-world data. Some stakeholders expressed their views on the cumulative burden of the MEAs on the healthcare system. Trust between stakeholders was also a concern that impacted implementation of MEAs. Given the complex process of designing and implementing the MEAs, the timing required to plan and implement it is also essential, to ensure timely access to these innovative treatments. Healthcare practitioners prefer a greater discount, whereas national payers prefer agreements which effectively manage the uncertainties. Manufacturers could additionally increase flexibility of their own budgets and educate policy makers to advocate health outcomes-based agreements. Properly incentivizing the stakeholders is also seen as enablers of MEAs implementation.

Looking at the implementation duration, stakeholders felt 2 to 5 years were an appropriate period for each MEAs. However, funding in the interim period of renegotiation, the administrative burden of renegotiation and the phasing out if renegotiation failed must be managed carefully to ensure continuity of treatment. Some system has passed on the cost during the renegotiation period to the pharmaceutical companies, while some shares the cost. Implementation of the MEAs also had to be ensured to cover appropriate disease areas, to ensure fairness of treatment. Availability of governance mechanisms in some countries is seen as best practices implemented due to long-standing experience with MEAs. Countries like China and Taiwan are encouraging innovations through drug pricing policies.

Ensuring the refund notification, rebates and reimbursements of MEAs are paid out in a timely and correct manner was also a concern among stakeholders, to ensure continued trust. Continuous process improvement, formalization of processes and early engagement could improve the overall implementation.

Stakeholders felt the availability of effective guiding framework for the implementation and delisting of MEAs were an important aspect for alignment between stakeholders, for patient data collection and negotiations. The framework would reduce lengthy negotiations as delays are mostly related to an unwillingness to compromise and the inability to agree on performance metrics. Absence of guiding frameworks are an important barrier to launch and operate MEAs. Majority of the countries which has implemented the MEAs has either laws, frameworks, regulation mechanism or as part the tendering process to implement and administer it. Though the frameworks are available, some can be manipulated, or some frameworks are created to address external pressure even when the health systems are not ready to implement it.

Stakeholder acceptance of the model and the provided budget impact estimates would depend on the numerical parameters and tolerance level imposed in the local MEAs mechanism. This also brings to light the need to study optimal behaviours of parties engaged in the MEAs [12].

### 3.6 Limitations of the reviewed studies

Study limitations were not explored in many of the studies. A few common issues were highlighted in some of the studies which discussed its’ limitations. Some of the relevant stakeholders could not participate in the studies due to confidentiality concerns. The other concern was the consenting respondents may be positively inclined towards the MEAs. In addition to that, the sampling technique where a purposive sampling also led to limited participation from certain stakeholders. The small sampled nature of the studies could lead to underestimation and fewer data learning points. Selection bias was observed in some studies, where only large pharmaceutical and patient advocacy organizations were sampled. Timeliness of the access schemes was not explored in most studies. Review criteria and duration were not extensively discussed. Some studies focussed on specific drug classes, where useful information may be omitted. Most articles documented the snapshot of the policymakers’ perceptions, however, the social desirability of MEAs were not explored. Complete data sets could not be obtained due to the confidential nature of the MEAs. Except one Australian study [11], none of the studies considered patients’ views on the MEAs, its’ benefit and challenges. Understanding the patients' views in terms of benefit, challenges and their level of participation/ contribution in the implementation could provide additional insights and improve its’ implementation.

### 3.7 Assumed future of MEAs

Lu CY et al. [11] explored the idea of government bodies and pharmaceutical organizations collaboratively funding registries and the consumer organizations disseminating information and providing support in its implementation. The use of health informatics was also seen as an avenue to bridge the data deficiency. The MEAs objectives should be explicit and transparent, where ethical governance should be managed by appropriate professionals [25]. Integrated evidence generation plan and incentivizing the stakeholders are other means of improving data collection [28]. Formalizing the implementation of the MEAs via legislation steps is also seen as a way forward [16], [18]. With the formalization, non-sensitive information should be made public. If the MEAs are practised in a localised manner without a cross-institutional work stream, it may not benefit the worldwide system [20], [21]. Based on the current financial limitations of the health system, the MEAs will increase in the future including the performance-based ones [27]. However, the limitations of implementation, especially confidentiality may limit its growth [13]. Other method of MEAs with flexible pricing and simple data collection is also seen as a potential method of improving access [28].

A standard scheme may not fit all types of drugs, diseases or health systems, thus different types of schemes including hybrid schemes may be considered for improved implementation of MEAs. This is due to the heterogenous nature of pricing and reimbursement practices across these countries. Most systems reported implementing finance-based schemes more commonly than the outcomes/performance-based ones due to the reduced administrative burden. Italy has the most outcomes/ performance-based arrangements follow by Australia, UK and Scotland. Most payers preferred the finance-based arrangements due to its' simple nature, where it delivers a specific value with a simple reduction in price. The larger European economies had more experience with MEAs in comparison to the central and eastern European countries. Central and eastern European countries took a more watchful approach and in line with the different levels of formalization of the MEAs legal framework. Smaller economies faced issues with the capability of their health system to cope with the demands of the complex arrangements, thus negating the option of using outcomes/ performance based MEAs.

Stakeholder choices are not clearly explained, as to the role of the stakeholders in the individual health systems and their role in the decision making or implementation process related to the MEAs. Identifying stakeholders is a challenge due to the varied structure of decision making in terms of reimbursements in different jurisdictions. Patients or patient representatives had a say in the MEAs only in a few countries like Australia and UK. Thus, it is important to identify the role played by the individual stakeholders in the MEAs, to weigh the depth of their views. Patient involvement at least in the final stage of negotiation or assessment should be considered as they bear the consequences of the decisions made.

Trust between stakeholders is a highlighted issue in a few of the articles. The trust issue may be overcomed by increased engagement and communication. Regularly raised concerns are of the true reasons for the initiation of MEAs, on whether it was intended to improve patient access, collecting more data or a method to gain early market access and profits. Initiation of the MEAs by the payers based on their disease and financial status would seek to alleviate the trust concerns. This way the payers could target the diseases which are impacting a specific population or consuming a certain portion of the budget. This initiative could also address the issue of lack of data accessibility to the pharmaceutical organizations. As the data owners, both in terms of outcome and finance, the payers would be in a better position to determine what will be the benefit of new innovative drugs being introduced into their health system. Joint scientific advice process via integrated- and real world- evidence generation also reduces the trust barriers. Payers and manufacturers should have a clear understanding of the clinical and economic uncertainties. Scarce information and publications on MEAs, its’ success and failure reduce the exposure of stakeholders in other jurisdiction, which could further delay its’ adoption and up-take upon adoption.

Use of trusted third party in structuring the arrangements, collecting and interpreting data is also seen as an option to ease implementation. The additional cost of evaluation is paid either by payers, manufacturers or both depending on arrangements. Centralised bodies of healthcare providers and independent health technology assessment organizations are seen to be able to develop, negotiate and assess the MEAs in line with their healthcare needs.

Lack of expertise is a major concern among most jurisdictions. The schemes should be evaluated by trained professionals. Sharing of application, negotiation and implementation framework among the payer organizations could reduce the knowledge gap. The availability of a regional patient access scheme/ pricing network among payers could be a basis of knowledge and expertise sharing minus the confidential information, could shorten the time to access for expensive medicines. Such collaboration can consolidate the bargaining power of the region to ensure patients’ timely access to medicines at affordable price. Payers should try to overcome the lack of transparency and information asymmetry to increase collaborations amongst them. This could also be beneficial for the suppliers due to reduced transaction time and costs as this would mitigate the need for separate negotiations. Transparency in terms of pricing also affects the international reference pricing, which leads to discontent among payers. This also leads to ‘whipsawing’ where when the manufacturers use the acceptance of certain jurisdictions to pressure others to also accept the technologies but on the different terms or arrangement as the information is confidential. Low transparency could not ensure fair use of public finances and widens the gap between society and government while leaving room for fraud. In view of the confidentiality, there is a need to carry out formal internal evaluations of the arrangements to ensure it’s beneficial to the health system. Transparency of at least certain information could also be included in the arrangements. Variations exists when it comes to sharing of information on MEAs. In some countries, it is strictly confidential. Some jurisdictions like Italy, Sweden, Scotland, England and Wales makes the existence of arrangement public, while keeping the financial information confidential. Italy allows use of its registry of arrangements without the pricing details, which allows the different regions to negotiate either at the national or the regional levels.

The political philosophy of the individual countries is also an important component in the push for the introduction of high-cost innovative medicines. The political philosophy can play a tremendous role by promoting research and development by building political commitment to drive research, innovations and policies that allow MEAs to flourish. There is also limited mention of political push to implement MEAs. However, increased adoption of MEAs are observed in countries with a higher political push such as US, UK, Germany, the Netherlands and Sweden. This however does not affect the challenges faced in its' implementation.

Common issues in all the published studies were objectives of the arrangements, needs and capacity of health systems to handle the arrangements, handling of post or the interim period of the arrangements and assessing the value of the arrangements to ensure the systems benefit from it.

### 3.8 Limitation

This literature review was based on available literature on the stakeholders’ view of both private and public sector use of MEAs that included both finance- and outcomes/ performance- based MEAs. Thus, the conclusion of this study reflectes both types of MEAs. As most jurisdictions did not elaborate on the process of the MEAs implementation, the appropriateness of the stakeholders’ types could not be ascertained. Lack of transparency in most health systems are also a hindrance to further understanding and confidence in the MEAs. The views of the stakeholders may also evolve with experience.

## 4 Conclusion

In conclusion, the use of managed entry arrangements is expected to grow as pressure mounts on the payer to provide access newer technologies and to address the unmet needs. Currently there are no alternative as manufacturers will not provide a transparent discount. Though there are challenges in the designing and implementation of MEAs, close networking, improved human capital development especially in the area of health technology assessment and health economics would be able to close the gap in the implementation. However, this may not be a sustainable solution in the long run if the gaps are not addressed adequately. Early dialogue will allow the pharmaceutical organisations to have a better understanding of the needs of payers, patients, and HTA bodies. Based on the current health system capabilities, most systems prefer a is simple arrangement or direct discounts, though this may not address the outcome uncertainties. A more transparent approach from all stakeholders and independent analysis of the benefits or potential benefits, both in terms of cost and outcomes is needed.

## CRediT authorship contribution statement

**Subramaniam Thanimalai:** Conceptualization, Methodology, Writing - original draft. **Choon Wai Yee:** Writing - review & editing, Validation, Supervision. **Kenneth Lee Kwing-Chin:** Conceptualization, Supervision, Writing - review & editing.

## Declaration of Competing Interest

The authors declare that they have no known competing financial interests or personal relationships that could have appeared to influence the work reported in this paper.
