# Supplementary material for: Stakeholder views of managed entry agreements: A literature review of national studies
Source: Health Policy Open. 2021 Jan 21;2:100032. doi: 10.1016/j.hpopen.2021.100032 (PMC10297797; doi:10.1016/j.hpopen.2021.100032)
Supplement: Supplementary data 1 [file mmc1.docx]

APPENDIX I: ‘SPIDER abstract/ title screening’

We used SPIDER (1, 2)to develop the screening tool, comprising 3 the following elements:

| Sample | Healthcare personnel and other individuals involved in impacting innovative patient access scheme, including;   - Pharmaceutical company personnel - Healthcare Service Provider/ Payer/ Insurance personnel - Patient Advocacy group personnel |
| --- | --- |
| Phenomenon of Interest | Healthcare personnel and other individuals’ perceptions and experiences regarding innovative patient access schemes. |
| Design | Data collection methods will include but not limited to interviews, focus group discussions, document analysis and observations  Data analysis methods will include but not limited to thematic analysis and grounded theory |
| Evaluation | Experiences and perceptions |
| Research Type | Qualitative |
